# Complete sequential assignment and secondary structure prediction of the cannulae forming protein CanA from the hyperthermophilic archaeon *Pyrodictium abyssi*

**DOI:** 10.1007/s12104-020-09934-x

**Published:** 2020-02-12

**Authors:** Raphael Kreitner, Claudia E. Munte, Katrin Singer, Karl O. Stetter, Gudrun Horn, Werner Kremer, Hans Robert Kalbitzer

**Affiliations:** 1 Institute of Biophysics and Physical Biochemistry, Biophysics I and Centre of Magnetic Resonance in Chemistry and Biomedicine (CMRCB), University of Regensburg, Universitätsstr. 31, 93053 Regensburg, Germany; 2 Lehrstuhl für Mikrobiologie und Archaeenzentrum, University of Regensburg, Universitätsstr. 31, 93053 Regensburg, Germany

**Keywords:** *Pyrodictium abyssi*, CanA, Cannulae, Extremophile, Solution NMR, Assignments

## Abstract

CanA from *Pyrodictium abyssi* forms a heat-resistant organic hollow-fiber network together with CanB and CanC. An N-terminally truncated construct of CanA (K_1_-CanA) gave NMR spectra of good quality that could be assigned by three-dimensional NMR methods on ^15^N and ^13^C–^15^N enriched protein. We assigned the chemical shifts of 96% of all backbone ^1^H^N^ atoms, 98% of all backbone ^15^N atoms, 100% of all ^13^C^α^ atoms, 100% of all ^1^H^α^ atoms, 90% of all ^13^C′ atoms, and 100% of the ^13^C^β^ atoms. Two short helices and 10 β-strands are estimated from an analysis of the chemical shifts leading to a secondary structure content of K_1_-CanA of 6% helices, 44% β-pleated sheets, and 50% coils.

## Biological context

The members of the archaeal family *Pyrodictiaceae* have typical growth temperatures between 75 and 110 °C (348 to 383 K) (Stetter 1982; Stetter et al. 1983; Pley et al. 1991). Three different species of *Pyrodictium* are known, *P. occultum, P. brockii* and *P. abyssi*. *P. abyssi* was isolated from a black smoker, a hydrothermal deep-sea vent 3600 m below sea level (Deininger 1994). As in most of the *Crenarchaeota* the surface of the cells is covered with a protein layer (S-layer) with hexagonal symmetry. A specific feature of the *Pyrodictiaceae* is the formation of a complex extracellular matrix connecting the cells. The matrix consists of interconnected hollow fibers (cannulae) consisting of helically arranged subunits (König et al. 1988; Rieger et al. 1995). The cannulae are formed from glycoproteins and connect the periplasmic space of different cells (Nickell et al. 2003). While the biological function of cannulae is still unknown, their arrangement suggests that they serve to the exchange of molecular components (metabolites, genetic information, signaling) between the cells in the network (Horn et al. 1999). Optical microscopy under *in-vivo* conditions shows that the polymerisation of these tubules is coupled to the cell division and the daughter cells stay connected after division. The tubules grow with a velocity of 1–1.5 µm/min (Horn et al. 1999). The heat stability of the cannulae is remarkable; even after exposure at 413 K for one-hour intact cannulae are found (Rieger et al. 1995). The cannulae consist of three helically arranged highly homologous glycoprotein subunits CanA, CanB, and CanC (Mai 1998). CanA is composed of 182 amino acids with a total molecular mass of 19.8 kDa. For the transport to the periplasm, a signaling sequence of 25 amino acids is required. No proteins with similar sequences are found in the protein databases. In *Escherichia coli*, expressed CanA monomers spontaneously form stable tubules with the same characteristics as native cannulae.

The heat resistance of the hollow-fibers promises interesting biotechnological applications. In nanotechnology, carbon nanotubes are a central research topic. CanA nanotubes could take a similar role in nanobiophysics, since they are heat stable, self-organizing, and can simply be modified by site-directed mutagenesis. A prerequisite for the structure determination of CanA is the complete sequential assignment of uniformly ^13^C–^15^N enriched protein that is presented here.

## Materials and experiments

### Protein expression and purification

**Fig. 1:**
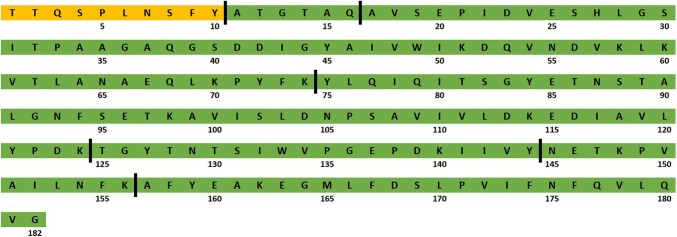
Amino acid sequence of CanA from *P. abyssi*. The sequence of K1-CanA is depicted in green. In orange, the additional amino acids of the full length CanA after removal of the signaling sequence are shown. The cleavage sites obtained by limited proteolysis of CanA with trypsin for 3 h are labeled with black bars

K_1_-CanA is an N-terminally truncated construct of CanA whose sequence is depicted in Fig. 1. This truncated version of CanA has been selected for assignments after limited proteolysis experiments (see below). It exhibits NMR spectra with much higher quality than the full-length protein.

The pET17b-CanA plasmid (Amp^r^, T7) contains the 549 bp long sequence for CanA without a 75 bp long leading sequence. The pET17b-K1CanA plasmid corresponds to the pET17b-CanA plasmid but with a coding sequence shortened by 30 bp. For obtaining unlabeled CanA or the N-terminally truncated K_1_-CanA *E. coli* BL21(DE3)pLysS transformed with pET17b-CanA and pET17b-K1CanA were grown overnight and then inoculated 1:100 into fresh LB-medium containing chloramphenicol (34 mg/L) and carbenicillin (50 mg/L), and grown at 310 K to an OD of 0.8 at 600 nm. Protein expression was induced by adding IPTG (final concentration 0.8 mM). After 16 h, the cells were harvested by centrifugation. To prepare uniformly ^15^ N- or ^13^C–^15^N-labeled proteins, cells were grown in isotopically labeled New Minimal Medium (NMM) (Budisa et al. 1995), pH 7.4, containing 1 g/L ^15^NH_4_Cl or 1 g/L ^15^NH_4_Cl and 2 g/L ^13^C-glucose, respectively. The yield of pure unlabeled CanA or K_1_-CanA was approximately 30 mg protein/L medium, of isotope labeled approximately 10 mg/L. Cells were centrifuged down, washed, and resuspended in 50 mM TRIS, pH 7.4, 50 mM NaCl, 5 mM EDTA. Cells were lysed by sonicating them 2-times for 30 s in an ice bath. Debris was spun down at 14 × 10^3^ g for 15 min at 277 K. The supernatant was shaken in a water bath with a temperature of 353 K for 15 min. After the heat denaturation, the sample was cooled in an ice bath for 10 min and the denatured protein was removed by centrifugation. The supernatant was transferred to Q-sepharose (HiLoad 26/10 Q Sepharose Fast Flow) column equilibrated with 50 mM TRIS–HCl, pH 7.4. The protein was eluted with a linear NaCl gradient using an Äkta chromatography system (ÄKTA™ FPLC + Frac-900) with flow rate of 4 mL/min and gradient mixing time (0 to 500 mM NaCl). All fractions were monitored by SDS/PAGE on 13% acrylamide gels. The fractions containing the protein were pooled where CanA (or K_1_-CanA) eluted at a NaCl concentration of approximately 150 mM. After concentrating the obtained CanA sample with a Vivaspin system (Sartorius Stedim Biotech, Göttingen) it was transferred to a gel filtration column (HiLoad 26/60 Superdex 75 prep grade) equilibrated with 50 mM TRIS/HCl pH 7.4, 50 mM NaCl and eluted isocratically. The purity of the sample was checked by SDS-PAGE, the integrity of the protein was confirmed by mass spectrometry. Amino acid sequencing showed that the N-terminal methionine residues of CanA and K_1_-CanA were removed by the expression system.

### Limited proteolysis of CanA

Samples of CanA were incubated for different times with 8 U trypsin (Merck, Darmstadt)/mg CanA at 310 K at pH 7.5. The obtained peptides were separated with SDS-PAGE and consecutively analysed by Edman degradation and N-terminal sequencing on a Procise 492A sequencer (Applied Biosystems) with on-line detection of the PTH (phenylthiohydantoin) amino acids. Their masses were determined by mass spectrometry. Already after 10 min, the first 10 amino acids were removed from CanA. This corresponds to the construct K_1_-CanA that is used for assignment purposes in this paper. Additional peptides were observed in the SDS-PAGE after 2 h incubation (molecular masses of approximately 15, 12 and 7 kDa) and after 66 h incubation (molecular masses of approximately 11, 6 and 4 kDa). Analysis of all 7 fragments extracted from the SDS-PAGE leads to 8 peptides with molecular masses of 18.7, 15.8 and 15.2, 12.2, 11.6, 6.7, 6.2, and 4.3 kDa. The cleavage sites obtained are indicated in Fig. 1. The cleavage sites located behind K124 and Y144 in the CanA sequence are part of a long region (V119–Y144) that is not present in the homologous proteins CanB and CanC. It does not seem to be important for the structural stability and is probably located on the surface of the protein.

### NMR spectroscopy

NMR experiments were carried out at 323 K on Bruker Avance 800 and Avance 600 spectrometers equipped with TCI and TXI cryoprobes and operating at proton resonance frequencies of 800.2 MHz and 600.1 MHz, respectively. NMR spectra were referenced to the methyl resonance of DSS used as internal standard. ^1^H resonances were referenced directly, ^13^C and ^15^N indirectly as defined by the IUPAC recommendations (Markley et al. 1998). Sequence-specific resonance assignments have been carried out on the basis of two-dimensional NOESY, TOCSY, [^1^H, ^13^C]-HSQC and [^1^H,^15^N]-HSQC experiments, and three-dimensional HNCO, HNCA, CBCA(CO)NH, CBCANH, HCAN, [^1^H, ^15^N]-NOESY-HSQC and HCCH-TOCSY experiments. Aromatic side chains were assigned with (HB)CB(CGCC-TOCSY)Har and (HB)CB(CGCD)HD experiments. Assignments were performed with the program AUREMOL (Gronwald and Kalbitzer 2004; https://www.auremol.de). Backbone dihedral angles and secondary structure propensities were predicted with the program TALOS-N (Shen and Bax 2013).

### Extent of assignment and data deposition

**Fig. 2:**
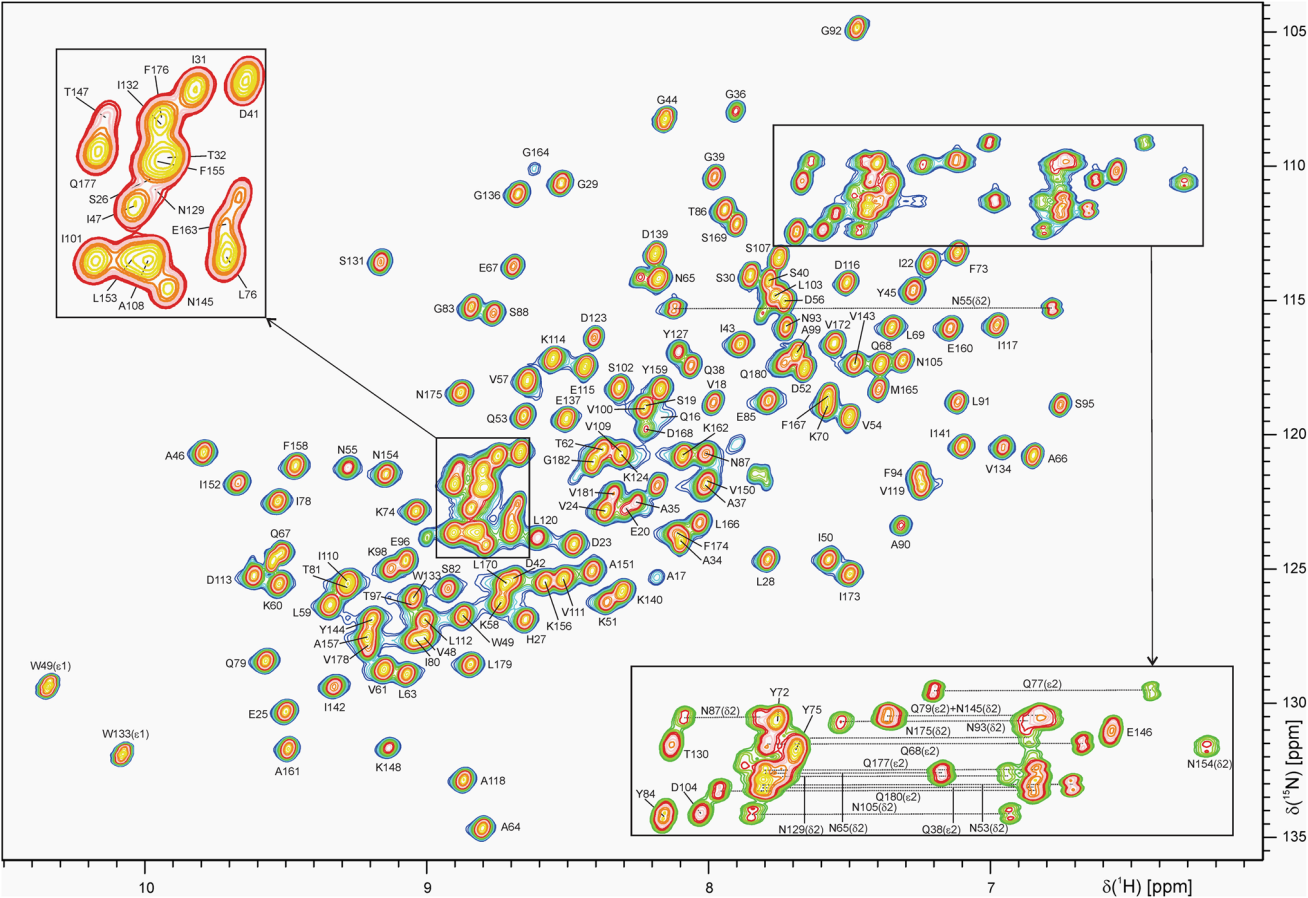
[^1^H, ^15^N]-HSQC spectrum of K_1_-CanA. 0.5 mM K_1_-CanA in 20 mM sodium phosphate buffer, pH 6.6, 0.1 mM EDTA, 0.4 mM NaN_3_, 0.4 mM DSS, 90% H_2_O/10% D_2_O. The [^1^H, ^15^N]-HSQC spectrum was recorded at a proton resonance frequency of 800.2 MHz at 323 K

The NMR data of K_1_-CanA were recorded typically at a protein concentration of 0.5 mM in 20 mM sodium phosphate buffer (Na_2_HPO_4_/NaH_2_PO_4_) pH 6.6 containing 0.1 mM EDTA, 0.4 mM NaN_3_, 0.4 mM DSS and either 90% H_2_O/10% D_2_O or 100% D_2_O, at 323 K. Only at this elevated temperature, spectra with that high quality could be obtained. Figure 2 shows an [^1^H, ^15^N]-HSQC-spectrum of K_1_-CanA at this temperature. Note that *P. abyssi* is growing in a temperature range between 340 to 378 K (“physiological temperature”) (Marteinsson et al. 1997).

We assigned the chemical shifts of 96% of all backbone ^1^H^N^ atoms, 98% of all backbone ^15^N atoms, 100% of all ^13^C^α^ atoms, 100% of all ^1^H^α^ atoms, 90% of all ^13^C′ atoms, and 100% of the ^13^C^β^ atoms. The side chain resonances were almost completely assigned including all aromatic side chains. Only the proton resonances of side chain amino groups of the 12 lysine residues were broadened beyond detection and could therefore not be assigned. Under favorable conditions, the configuration of the peptide bond in front of proline residues can be determined from the chemical shift difference ∆_βγ_ of the ^13^C^β^ and ^13^C^γ^ resonances For the *trans-* and *cis-*isomers one obtains ∆_βγ_ values of 4.51 ± 1.37 and 9.64 ± 1.62 ppm, respectively (Schubert et al. 2002). For 7 of the 9 proline residues the trans-configuration can be confirmed by this method, for P135 and P149 the method cannot be used since the ^13^C^γ^ resonances could not be identified (Table 1). Alternatively, the *cis–trans* isomers can be identified by sequential NOEs between the amino acid X preceding the proline residue and the proline. The *trans*-isomer is characterised by strong H^α^_(X)_–H^δ^*_(Pro)_ and H^N^_(X)_–H^δ^*_(Pro)_ NOEs, the *cis*-isomer by strong H^α^_(X)_–H^α^_(Pro)_ and H^N^_(X)_–H^α^_(Pro)_ NOEs (Wüthrich et al. 1984). With these data, the conclusions from the chemical shift analysis could be confirmed. Stereospecific assignments of side chain amide protons of asparagine and glutamine residues were performed with the program AssignmentChecker contained in AUREMOL that is based on the chemical shift difference between the two geminal protons (Harsch et al. 2017). Asparagine and glutamine amide proton resonances can be stereochemically assigned if chemical shift differences are ≥ 0.40 ppm for asparagine and ≥ 0.42 ppm for glutamine with a confidence level > 95%. In this case, the downfield shifted resonance lines can be assigned to H^δ21^ and H^ε21^, respectively. These assignments were confirmed, if possible, by the analysis of the corresponding NOEs (Wüthrich 1986). In this manner, the side chain amide protons stereospecific assignment of all 8 glutamine and of 7 from 9 asparagine could be performed. In one asparagine (N154) the amide proton resonances are degenerate and their mean is shifted significantly upfield (6.30 ppm). The resonance assignments were deposited in the BioMagResBank (https://www.bmrb.wisc.edu) under accession number 50124.

**Table 1:** Configuration of the peptide bond in front of proline residues^a^

Amino acid	C^β^ (ppm)	C^γ^ (ppm)	Δ_βγ_ (ppm)	Strong NOE	Isomer
P21	32.11	27.27	4.84	–^b^	trans
P33	31.65	26.96	4.69	H^α^(32)–H^δ^(33)	trans
P71	31.33	27.74	3.59	H^N^(70)–H^δ^(71)	trans
P106	32.74	26.49	6.25	H^α^(105)–H^δ^(106)	trans
P122	32.27	26.80	5.47	–^b^	trans
P135	31.33	–^c^	−	–^b^	?
P138	31.18	26.80	4.38	H^α^(137)–H^δ^(138)	trans
P149	31.78	–^c^	−	–^b^	?
P171	32.43	26.33	6.10	H^α^(170)–H^δ^(171)	trans

^a^The peptide bond configuration was determined from the chemical shift difference ∆_βγ_ of the C^β^ and C^γ^ shifts of the proline residues according to (Schubert et al. 2002) and/or from the NOEs between the amino acid X preceding the proline residue and the proline residue according to Wüthrich et al. (1984)

^b^Resonances could not be assigned

^c^No unambiguous NOEs could be found

### Secondary structure prediction

**Fig. 3:**
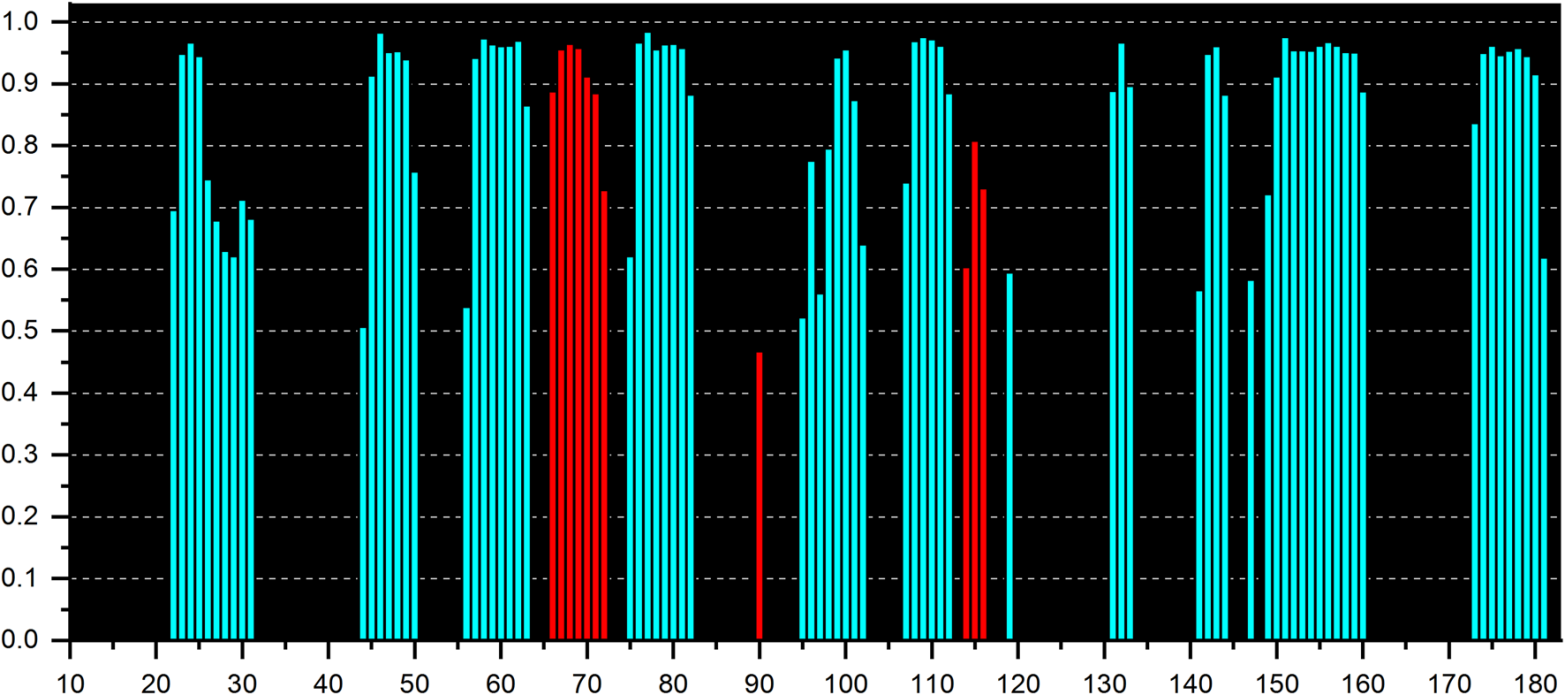
Secondary structure propensities of K_1_-CanA from chemical shifts. The secondary structure propensities were obtained from the chemical shifts of C', C^α^, C^β^, N, H^N^ and H^α^ atoms by the program TALOS-N (Shen and Bax 2013), (blue) propensities for β-strands, (red) propensities for helices

From an analysis of the C′, C^α^, C^β^, N, H^N^ and H^α^ chemical shifts by the program TALOS-N the secondary structure propensities can be estimated. They are depicted in Fig. 3 showing high propensities for two helices and 10 β-strands. The corresponding regions in the sequence are presented in Table 2. In total, from the chemical shifts a secondary structure content of K_1_-CanA of 6% helices, 44% β-pleated sheets and 50% coils is predicted. Since the first 10 amino acids of CanA most probably are also disordered, similar values are obtained for the complete protein (6% helices, 41% β-pleated sheets and 53% coils). These experimental values are surprisingly close to the secondary structure prediction obtained by PredictProtein (https://www.predictprotein.org) (Rost et al. 2004) that predicts 7% α-helices, 43% β-pleated sheets and 50% coils from the amino acid sequence.

**Table 2:** Location of secondary structure elements in K_1_-CanA

Secondary structure	Amino acid	Helix	Strand	Coil
L1	11–21			×
E1	22–31		×	
L2	32–43			×
E2	44–50		×	
L3	51–55			×
E3	56–63		×	
L4	64–65			×
H1	66–72	×		
L5	73–74			×
E4	75–82		×	
L6	83–94			×
E5	95–102		×	
L7	103–106			×
E6	107–112		×	
L8	113			×
H2	114–116	×		
L9	117–130			×
E7	131–133		×	
L10	134–140			×
E8	141–144		×	
L11	145–148			×
E9	149–160		×	
L12	161–172			×
E10	173–181		×	
L13	182			×

The secondary structure propensities were obtained from the chemical shifts of C', C^α^, C^β^, N, H^N^ and H^α^ atoms by the program TALOS-N (Shen and Bax 2013)

*Li* coil regions, *Ei* β-strand regions, *Hi* helix

## Abbreviations

CD: Circular dichroism
DSS: 2,2-Dimethyl-2-silapentane-5-sulfonate
DTE: 1,4-Dithioerythritol
GdmCl: Guanidinium hydrochloride
